# ADIPOR1 deficiency-induced suppression of retinal ELOVL2 and docosahexaenoic acid levels during photoreceptor degeneration and visual loss

**DOI:** 10.1038/s41419-021-03741-5

**Published:** 2021-05-07

**Authors:** Hideto Osada, Eriko Toda, Kohei Homma, Naymel A. Guzman, Norihiro Nagai, Mamoru Ogawa, Kazuno Negishi, Makoto Arita, Kazuo Tsubota, Yoko Ozawa

**Affiliations:** 1 Laboratory of Retinal Cell Biology, Tokyo, Japan; 2 Department of Ophthalmology, Keio University School of Medicine, 35 Shinanomachi, Shinjuku-Ku, Tokyo, 160-8582 Japan; 3 Laboratory for Metabolomics, RIKEN Center for Integrative Medical Sciences (IMS), 1-7-22, Suehiro-Cho, Tsurumi, Yokohama, Kanagawa 230-0045 Japan; 4 Division of Physiological Chemistry and Metabolism, Graduate School of Pharmaceutical Sciences, Keio University, 1-5-30, Shibakoen, Minato-Ku, Tokyo, 105-8512 Japan; 5 Cellular and Molecular Epigenetics Laboratory, Graduate School of Medical Life Science, Yokohama City University, Tsurumi, Yokohama, Kanagawa 230-0045 Japan; 6 Department of Ophthalmology, St. Luke’s International Hospital, 9-1 Akashi-Cho, Chuo-Ku, Tokyo, 104-8560 Japan; 7 Laboratory of Retinal Cell Biology, St. Luke’s International University, 9-1 Akashi-Cho, Chuo-Ku, Tokyo, 104-8560 Japan

**Keywords:** Mechanisms of disease, Developmental neurogenesis

## Abstract

Lipid metabolism-related gene mutations can cause retinitis pigmentosa, a currently untreatable blinding disease resulting from progressive neurodegeneration of the retina. Here, we demonstrated the influence of adiponectin receptor 1 (ADIPOR1) deficiency in retinal neurodegeneration using *Adipor1* knockout (KO) mice. *Adipor1* mRNA was observed to be expressed in photoreceptors, predominately within the photoreceptor inner segment (PIS), and increased after birth during the development of the photoreceptor outer segments (POSs) where photons are received by the visual pigment, rhodopsin. At 3 weeks of age, visual function impairment, specifically photoreceptor dysfunction, as recorded by electroretinography (ERG), was evident in homozygous, but not heterozygous, *Adipor1* KO mice. However, although photoreceptor loss was evident at 3 weeks of age and progressed until 10 weeks, the level of visual dysfunction was already substantial by 3 weeks, after which it was retained until 10 weeks of age. The rhodopsin mRNA levels had already decreased at 3 weeks, suggesting that reduced rhodopsin may have contributed to early visual loss. Moreover, inflammation and oxidative stress were induced in homozygous KO retinas. Prior to observation of photoreceptor loss via optical microscopy, electron microscopy revealed that POSs were present; however, they were misaligned and their lipid composition, including docosahexaenoic acid (DHA), which is critical in forming POSs, was impaired in the retina. Importantly, the expression of *Elovl2*, an elongase of very long chain fatty acids expressed in the PIS, was significantly reduced, and lipogenic genes, which are induced under conditions of reduced endogenous DHA synthesis, were increased in homozygous KO mice. The causal relationship between ADIPOR1 deficiency and *Elovl2* repression, together with upregulation of lipogenic genes, was confirmed in vitro. Therefore, ADIPOR1 in the retina appears to be indispensable for ELOVL2 induction, which is likely required to supply sufficient DHA for appropriate photoreceptor function and survival.

## Introduction

Recent progress in health research has revealed the significant impact of abnormal lipid metabolism in the pathogenesis of various diseases. Specifically, metabolic syndrome is associated with excessive intake of dietary lipids^1^; neural degenerative diseases, such as Alzheimer’s and Parkinson’s diseases, are related to oxidative stress-induced lipid peroxidation^2^ and retinal degenerative and blinding diseases, age-related macular degeneration^3^, as well as retinitis pigmentosa^4,5^, are reportedly caused by abnormal lipid accumulation.

Here, we focused on abnormal lipid metabolism in the retina, which is associated with adiponectin receptor 1 (ADIPOR1) deficiency. ADIPOR1 was initially described as an adiponectin receptor that affects systemic lipid and glucose metabolism^6–8^. However, more recently, it has also been described as a receptor for C1q tumor necrosis factor-related protein 9 (CTRP9), a newly discovered adipokine, which binds to SIRT1, a longevity factor^9^. Moreover, in humans, *ADIPOR1* mutation causes retinitis pigmentosa with or without systemic disorders, such as developmental and speech delays^10,11^.

Lipids serve as a major component of cellular membranes, while 30–60% of the total brain weight comprises lipids^2^. Similar to the brain, the retina is also a neural tissue that contains abundant lipid bilayers forming photoreceptor outer segment (POS) discs where light stimuli are received. Moreover, the function of the visual pigment, rhodopsin, a membrane-bound protein present in POS discs, is sensitive to the proportion of docosahexaenoic acid (DHA)-containing phospholipids present in the membrane^12,13^.

DHA is transported from the liver to the choroidal vessels, located beneath the retinal pigment epithelium (RPE)^14^, and through the RPE, subsequently transferred to photoreceptors, where it is used for the biogenesis of POS disc membranes as a major acyl chain^15^. ADIPOR1 deficiency reportedly reduces DHA uptake from the circulation to the photoreceptors^16^, leading to a reduction in DHA in the outer retina^17^. However, DHA (C22:6n-3) can also be locally produced by elongating the n-3 fatty acid precursors^18^ using very-long-chain fatty acid elongase (ELOVL) enzymes. These enzymes contribute to the maintenance of DHA levels in the brain, and impaired chain elongation causes neuroinflammation and demyelination^19^. Thus, a similar pathway for local DHA production could exist within the retinal tissue.

Here, we sought to characterize the mechanism associated with visual loss in an *Adipor1* KO mouse model by analyzing the molecular changes that occur during the development of the disorder. We ultimately propose that ADIPOR1 deficiency affects the expression of *Elovl2*, which contributes to DHA synthesis^18^, and leads to insufficient levels of DHA in the retina, which likely contributes to retinal neural degeneration in *Adipor1* KO mice. The current study will help elucidate the roles of ADIPOR1 in lipid metabolism and may contribute to the future exploration of a new therapeutic approach for retinal neurodegeneration.

## Materials and methods

### Animals

*Adipor1* KO mice (Adipor1^tm1Dgen^) were originally produced by Deltagen (San Mateo, CA, USA) and delivered through Mutant Mouse Regional Resource Centers and the Jackson Laboratory (#005775). The *Adipor1* KO mice were provided as the 129P2/OlaHsd **×** C57BL/6 background and backcrossed with C57BL/6 mice more than 10 times before the experiments. BALB/c mice were purchased from CLEA Japan (Tokyo, Japan). All animal experiments were performed using male mice, except for photopic ERG for which both male and female mice were used, and conducted in accordance with the Association for Research in Vision and Ophthalmology Statement for the Use of Animals in Ophthalmic and Vision Research after approval by the Animal Studies Committee of Keio University School of Medicine (Approval number; 09203). Mice were anesthetized with intraperitoneal injection of combined anesthetics (midazolam 4 mg/kg BW [Sandoz Japan, Tokyo, Japan], medetomidine 0.75 mg/kg BW [Nippon Zenyaku Kogyo Co., Ltd., Fukushima, Japan], and butorphanol tartrate 5 mg/kg BW [Meiji Seika Pharma Co., Ltd., Tokyo, Japan])^20^. The samples were collected by sacrificing the minimum number of animals needed to ensure that the derived data would be constant and significant. Data collection and analyses were performed under genotype blinded conditions.

### In situ hybridization

Eyes were enucleated, embedded in OCT compound (Sakura Finetek, Tokyo, Japan), and immediately flash frozen. Cryosections (10 μm) were fixed with 4% paraformaldehyde, treated with 10 mg/ml proteinase K (Roche Applied Science, Penzberg, Germany), and hybridized with sense or antisense digoxigenin-labeled RNA probes at 65 °C overnight. Signals were detected using an alkaline phosphatase-conjugated anti-DIG antibody (Roche Applied Science, #11093274910, 1:1000) and NBT/BCIP (Roche Applied Science). Probes were generated by reverse transcription-polymerase chain reaction (PCR) using the following primers (5ʹ to 3ʹ) and a DIG RNA labeling kit (Roche Applied Science). Adipor1F: AGGGACTTCCCAAGTGCTTT, Adipor1R: CATAGCAGTGATAGCAGGTTTCTT, Elovl2F: AGACTCGAGAGCATTTAAGCGGGATCCTT, Elovl2R: TCTGAATT CCACAGTTCTGAGCAGGGACA.

### Electroretinography

ERG recording was performed as previously described^20–22^. Briefly, mice were dark-adapted for 12 h and placed under dim-red illumination until they were anesthetized. The ground and reference electrodes were placed on the tail and in the mouth, respectively, while the active gold wire electrodes were placed on the cornea. Full-field scotopic ERGs were recorded in response to a flash stimulus at intensities ranging from −2.1 to 2.9 log cd s/m^2^, and photopic ERGs were recorded after 10 min of light adaptation in response to flash stimuli ranging from 0.6 to 1.6 log cd s/m^2^ with a background of 30 cd s/m^2^ (Ganzfeld System SG-2002; LKC Technologies, Inc., Gaithersburg, MD, USA), using PowerLab System 2/25 (AD Instruments, New South Wales, Australia) after pupil dilation with a mixture of 0.5% tropicamide and 0.5% phenylephrine (Mydrin-P, Santen Pharmaceutical, Osaka, Japan). The responses were differentially amplified and filtered through a digital bandpass filter ranging from 0.3 to 450 Hz. The a-wave amplitude was measured from baseline to trough, whereas the b-wave amplitude was measured from the a-wave trough to the b-wave peak. The implicit times were measured from stimulus onset to the peak of each wave. Peak points were automatically indicated by the system and confirmed by the examiner.

### Histological analyses

Mouse eyes were enucleated and fixed in 4% paraformaldehyde. Paraffin (Sakura Finetek Japan) sections (8 μm), including the optic nerve head to the most peripheral region of the retina, were deparaffinized. After staining with hematoxylin and eosin, outer nuclear layer (ONL) thickness was measured using ImageJ (developed by Wayne Rasband, National Institutes of Health, Bethesda, MD, USA; available at http://rsb.info.nih.gov/ij/index.html) 500 μm from the optic nerve head. Terminal deoxynucleotidyl transferase-mediated deoxyuridine triphosphate nick-end labeling (TUNEL) was performed using the ApopTag red apoptosis detection kit (Millipore, Bedford, MA, USA). For immunostaining, the sections were incubated with anti-glutamine synthetase (GS; BD Biosciences, Franklin Lakes, NJ, USA; #610158, 1:500), or anti-glial fibrillary acidic protein (GFAP; DAKO, Santa Clara, CA, USA, #Z0334, 1:500) antibodies; signals were obtained using Alexa 488-conjugated goat anti-rabbit IgG and Alexa 555-conjugated goat anti-mouse IgG, respectively. Nuclei were stained with Cellstain-4′,6-diamidino-2-phenylindole solution (Dojindo Molecular Technologies, Kumamoto, Japan, 2 μg/mL). Fluorescent images were obtained using a confocal microscope (TCS-SP5; Leica, Tokyo, Japan). TUNEL-positive cells in each section were counted and averaged.

### Immunoblot analyses

Samples were separated by sodium dodecyl sulfate-polyacrylamide gel electrophoresis, and proteins were transferred to a polyvinylidene fluoride membrane (Immobilon-P; Millipore). The membrane was blocked with 0.5% tyramide signal amplification blocking reagent (PerkinElmer Life Sciences, Waltham, MA, USA), incubated with anti-rhodopsin (Cosmo Bio Co., Ltd., Tokyo, Japan, #LSL-LB-5597-EX, 1:1,000,000), or anti-a-tubulin (Cell Signaling Technology, Danvers, MA, USA; #2125, 1:5,000) antibodies, and subsequently incubated with horseradish peroxidase-conjugated secondary antibody (Jackson Immuno Research Laboratories Inc., West Grove, PA, USA). Signals were detected using ECL Western Blotting Detection Reagents (GE Healthcare Limited, Buckinghamshire, UK), through a digital imager (LAS-4000 mini, GE healthcare), and their intensities were quantified using ImageJ and normalized to α-tubulin.

### Real-time reverse-transcription PCR

Total RNA was isolated with TRIzol reagent (Life Technologies, Carlsbad, CA, USA) and reverse transcribed using the SuperScript VILO master mix (Life Technologies). Real-time PCR was performed using the StepOnePlus™ PCR system (Applied Biosystems, Foster City, CA, USA), and gene expression was quantified using the 2^-ΔΔCT^ method and normalized to *Gapdh*^20,21^. Primers are listed in Table 1.

**Table1:** Primer list for real time PCR.

*Gene*	Forward primer (5′–3′)	Reverse primer (3′–5′)
*Gapdh*	AGGAGCGAGACCCCACTAAC	GATGACCCTTTTGGCTCCAC
*Rhodopsin*	AACTTCGGCCCCATCTTCA	CAGTGGATTCTTGCCGCAG
*Adipor1*	ATGGGGCTCCTTCTGGTAAC	ACCTCCTCCTCCTCTTCCTGA
*Crx*	ACCCAGTACCCGGATGTGTA	TCGCCCTACGATTCTTGAAC
*Nrl*	GGAAGGGCCTCTTGGCTAC	GTTCAACTCGCGCACAGAC
*F4/80*	CTGTAACCGGATGGCAAACT	ATGGCCAAGGCAAGACATAC
*Ho-1*	ACGCATATACCCGCTACCTG	CCAGAGTGTTCATTCGAGCA
*Elovl1*	CTGATGTCTGGTTGGCTGAG	CCTTGGAAAGCATGAAGAGC
*Elovl2*	CACACAGGCTCAGCTGGTGCA	CCAAAGGGGAAGCCACAGGGC
*Elovl4*	TTCTATCGCTGGACCTGGAC	GAGCGTGCTTATGCTTATCG
*Elovl5*	CCATCCCGTCCATGCGTCCCTA	CCCCGCAGGTCGTCTGGATGA
*Elovl6*	GAGAACGAAGCCATCCAATG	GCCGACCACCAAAGATAAAG
*Elovl7*	ACTCAAGAAAGCGATGATAACG	GTACCCCAGCCAGACATCAC
*Hsd17b12*	TTAATGTGCTTTCCGTTTGC	CACTGGCTGAGGAGATGTTG
*Hacd1*	AGACGCCTAGGCTTACTGG	CGTACCATAGCAATAGCAAGAAC
*Hacd2*	TTCTTTCCAGGTGATGTCAAGA	TCCAGGCAATGACAAACAGA
*Hacd3*	AGACCTTGTGAAGCCAGAGC	TCTTTCCCACCAATGACTCC
*Hacd4*	CCCTTTTGGAACTTCTGCAC	TGATCACCCCAAAGAGAATG
*Srebf1*	CAGCAGCCCCTAGAACAAAC	GCTGTCAGCAGCAGTGAGTC
*Srebf2*	GCAGCAACGGGACCATTCT	CCCCATGACTAAGTCCTTCAACT
*Insig1*	TGCAGATCCAGCGGAATGT	CCAGGCGGAGGAGAAGATG
*Fdft1*	ATGGAGTTCGTCAAGTGTCTAGG	CGTGCCGTATGTCCCCATC
*Cyp51*	GACAGGAGGCAACTTGCTTTC	GTGGACTTTTCGCTCCAGC
*Fas*	CCAGGAGAGACTGTCACAAGG	ACTGGGATCCTCTGAACACCT
*Scd1*	TTCTTGCGATACACTCTGGTGC	CGGGATTGAATGTTCTTGTCGT
*Scd2*	GCATTTGGGAGCCTTGTACG	AGCCGTGCCTTGTATGTTCTG
*Mfsd2a*	GGGCTTCTGCATTAGCAAGT	TCAGGCACAAACCAGATGAG
*Gnat1*	ATGCCCGCACTGTGAAACT	CATTCCTCCAGCGAATACCC
*Pde6b*	GAAGCCCAGCCATACTCAGG	TCTTCTTTGCCGTGGAGGA
*Arr3*	AGCCCATTGATGGAGTCGTC	ATCACGGCCATAGCGAAAAG
*Pde6c*	AGGAGGTCCTTGCTGTGGTC	GGTGTGCTGCAGCCTTAGTG

### Electron microscopy

Mice were euthanized, and their eyes were immediately fixed with 2.5% glutaraldehyde, post-fixed in 2% osmium tetroxide, dehydrated in a series of ethanol and propylene oxide solutions, and embedded in epoxy resin. Sections were stained with uranyl acetate and lead citrate and examined and photographed using an electron microscope (model 1200 EXII; JEOL, Tokyo, Japan).

### Lipidomics

Lipidomics were performed as previously described^23,24^. Briefly, mouse retinas were frozen immediately after dissection and stored at −80 °C. Lipids were extracted by homogenizing the retina in a bead pulverizing machine (6500 rpm for 15 s, ×2), and the supernatant was applied for liquid chromatography–mass spectrometry (LC–MS)/MS analysis using a quadrupole time-of-flight mass spectrometer (TripleTOF 6600; Sciex, Framingham, MA, USA) equipped with an ACQUITY UPLC BEH C18 column (1.0 × 150 mm, 1.7-µm particle size; Waters, Milford, MA, USA). Data analysis was performed as previously described^25^.

### Cell culture

The mouse cell line, bEnd.3, (gifted by Dr. Miayyad Al-Ubaidi, University of Oklahoma, OK, USA) was maintained in Dulbecco’s modified Eagle’s medium (#08456-65; Nacalai tesque, Kyoto, Japan) supplemented with 10% fetal bovine serum (Life Technologies), 100 unit/ml penicillin, and 100 μg/ml streptomycin (Sigma-Aldrich, St. Louis, MO, USA). Either control siRNA (Negative Control Lo GC, Thermo Fisher Scientific, Waltham, MA, USA), or *Adipor1* siRNA (mouse Adipor1 MSS231725, Thermo Fisher Scientific) was introduced using Lipofectamine RNAiMAX Reagent (Thermo Fisher Scientific), according to the manufacturer’s protocol, and incubated for 24 h. Knockdown (KD) was confirmed using real-time PCR.

### Bisulfite PCR and efficiency of bisulfite modification

Twenty-four hours after siRNA transfection, DNA was extracted from bEnd.3 cells with NucleoSpin Tissue kit (Macherey-Nagel, Germany). Bisulfite conversion was performed using EZ DNA Methylation-Gold Kit (Zymo Research, Orange, CA). CpG island prediction and methylation primer design were performed using MethPrimer^26^. PCR was carried out in a final volume of 50 μl containing 100 ng bisulfite-treated DNA, 2.5 mM MgCl_2_, 400 nmol each primer, 0.3 mM each dNTP, 0.25 μl TaKaRa EpiTaq^™^ HS (for bisulfite-treated DNA) (1.25 U/50 μl). PCR cycling conditions were as follows: 40 cycles of 10 s at 98 °C, 30 s at 56 °C for, 90 s at 72 °C and finally ended with 1 min at 72 °C for an extension. PCR products were separated on 1.0% agarose gel, purified using QIAquick Gel Extraction Kit (QIAGEN, Hilden, Germany), and then cloned into the pMD20-T vector using Mighty TA-cloning Kit (Takara, Japan). At least ten positive recombinant colonies of each product were sequenced by the Sanger method (Eurofins Genomics, Japan). The analysis of 65 bisulfite sequences was carried out with the QUMA (QUantification tool for Methylation Analysis) program (http://quma.cdb.riken.jp).

### Statistical analysis

Data are expressed as means ± standard deviations. Statistical analyses were performed using one-way analysis of variance with Tukey’s post hoc tests for comparisons among three or more groups or two-tailed Student’s *t* tests or Fisher’s exact test for comparisons between two groups using SPSS Statistics 26 (IBM, Armonk, NY, USA). Differences were considered statistically significant at *P* < 0.05.

## Results

### *Adipor1* expression in the retina

**Fig. 1:**
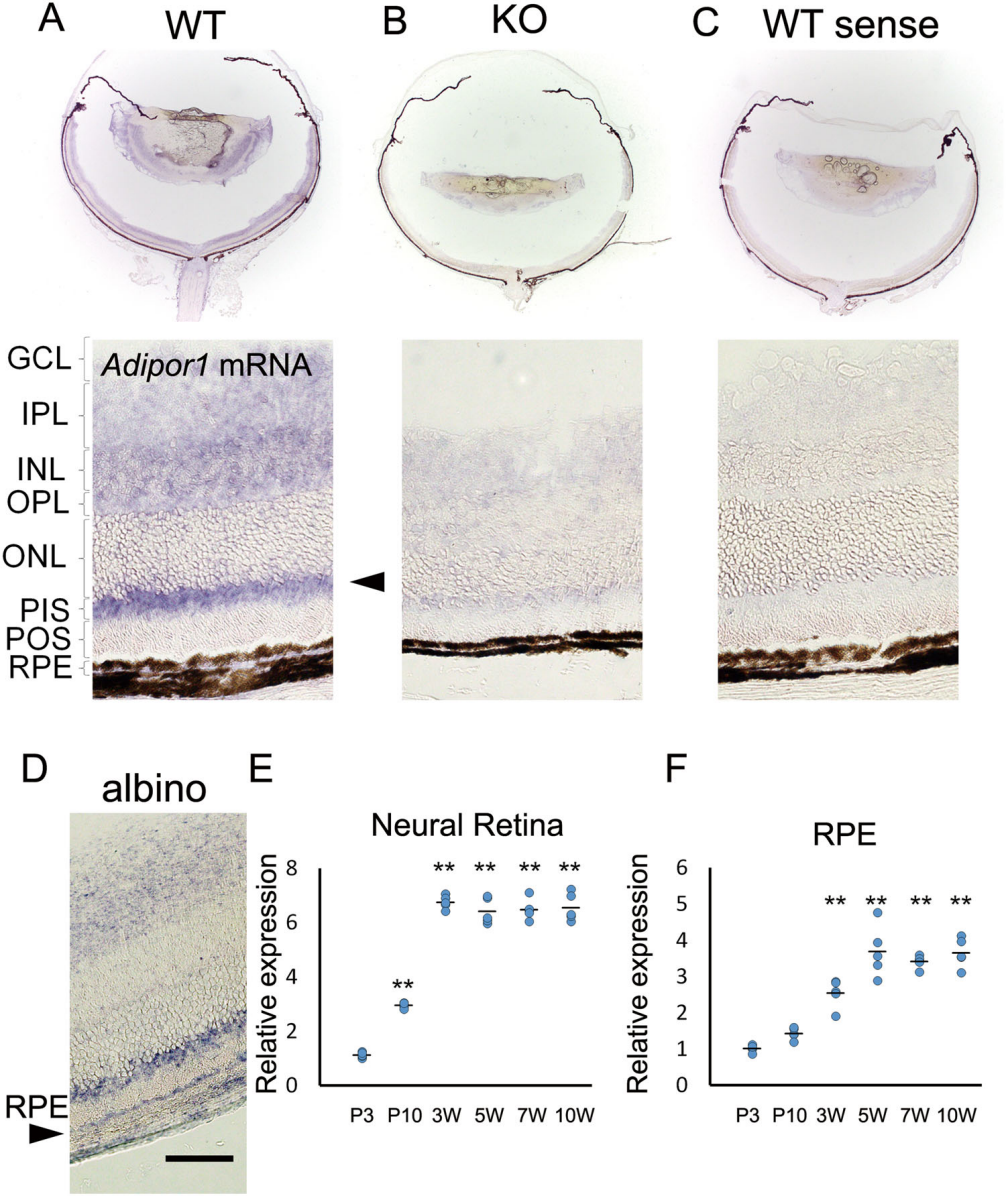
*Adipor1* expression in the retina. **A**–**D** In situ hybridization. *Adipor1* mRNA was prominently expressed in the PISs (arrowhead) and weakly in the inner retinal layers of WT mouse retina at 4 weeks of age (**A**). No signals were recorded with the antisense probe in the *Adipor1* KO retina (**B**) or the sense probe in the WT retina (**C**). Note that retinal thinning had already begun in the KO mice at this time point (see Fig. 3 for details). *Adipor1* mRNA was also expressed in the RPE (arrowhead) as shown in BALB/c albino retinas (**D**). **E**, **F** Real-time PCR. *Adipor1* mRNA increased during retinal development, peaking at 3 weeks of age in the neural retina (**E**) and at 5 weeks of age in the RPE (**F**). GCL ganglion cell layer, INL inner nuclear layer, IPL inner plexiform layer, ONL outer nuclear layer, OPL outer plexiform layer, PIS photoreceptor inner segment, POS photoreceptor outer segment, RPE retinal pigment epithelium. Data are shown as means ± standard deviations. *n* = 5, **P < 0.01 vs. P3, one-way ANOVA.

*Adipor1* mRNA was prominently expressed in the photoreceptor inner segments (PIS), and in the inner layers of the wildtype (WT) retina at 4 weeks of age (Fig. 1A). No specific signals were detected in *Adipor1* KO retinas with the antisense probe (Fig. 1B) or WT retinas hybridized with the sense probe (Fig. 1C). As pigments that interfere with mRNA detection in the RPE are present in C57B/6 WT mice, albino BALB/c mice were analyzed to demonstrate *Adipor1* mRNA expression in the RPE (Fig. 1D). *Adipor1* mRNA was detected in the neural retina by postnatal day 3 (P3), and the expression increased by P10, reaching a plateau at 3 weeks, which was maintained thereafter (Fig. 1E). In the RPE, the levels had upregulated by 3 weeks, peaking at 5 weeks, and were maintained thereafter (Fig. 1F).

### *Adipor1* KO mice had visual function impairment

**Fig. 2:**
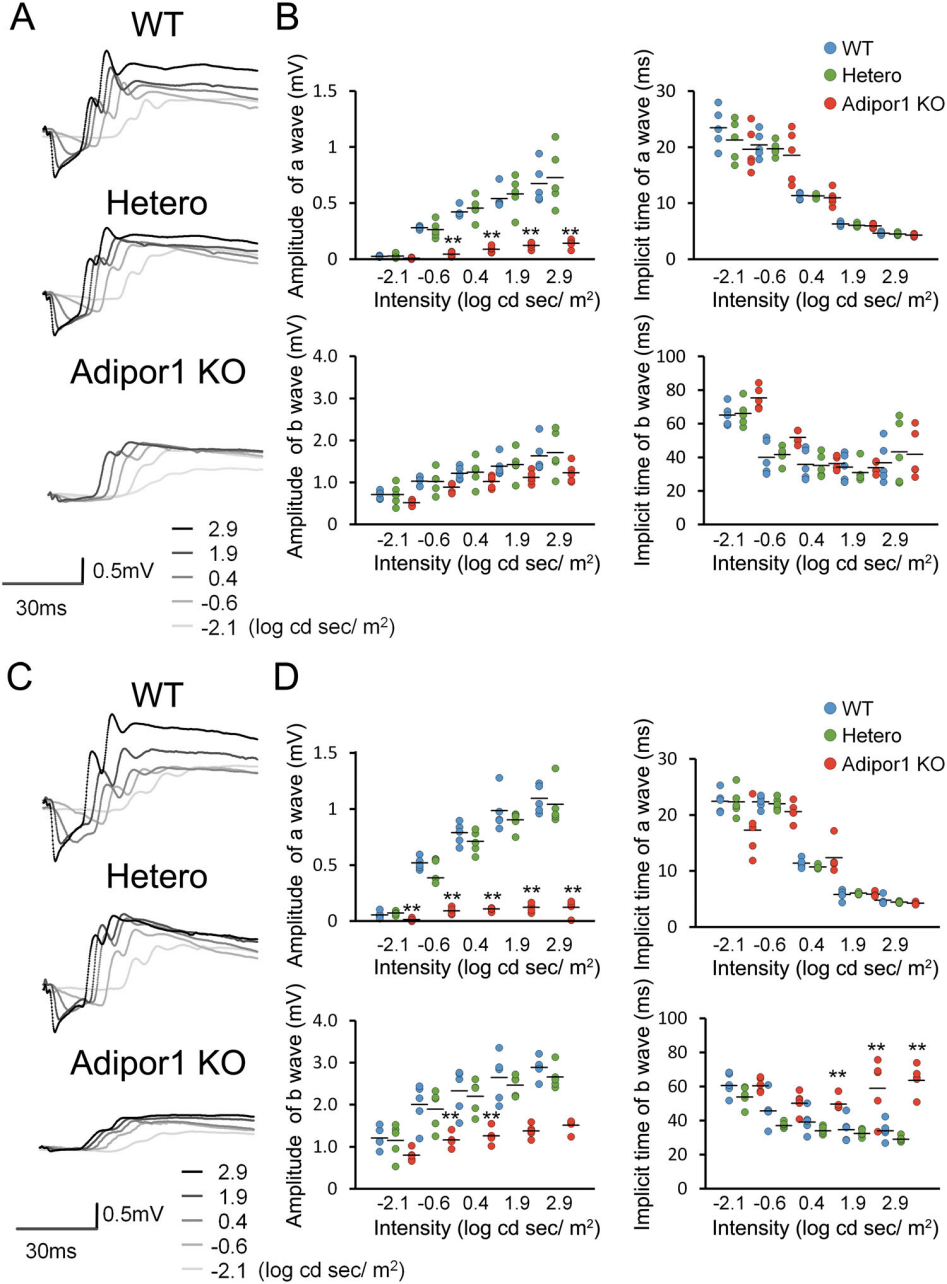
Visual function impairment in homozygous *Adipor1* KO mice. Full-field scotopic ERGs at 3 (**A**, **B**), and 10 weeks of age (**C**, **D**). Representative waveform from individual mice at each stimulus intensity (**A**, **C**). The a-wave amplitudes decreased in homozygous KO mice compared to WT and heterozygous mice at 3 and 10 weeks. The reduction in amplitudes and increased implicit time of the b-wave became evident at 10 weeks. No differences were observed between WT and heterozygotes. Data are shown as means ± standard deviations. *n* = 5, **P < 0.01 vs. WT, one-way ANOVA.

Scotopic ERG revealed a marked decrease in the a-wave amplitude representing photoreceptor function in the homozygous *Adipor1* KO mice at 3 weeks of age (Fig. 2A, B), although no changes were observed in heterozygous mice. The amplitudes of the a-wave and of the b-wave in KO mice at 10 weeks were comparable to those at 3 weeks; however, the implicit time of the b-wave, which reflects subsequent neuronal network function to the photoreceptors, was increased at 10 weeks (Fig. 2C, D). Thus, visual function, particularly, rod photoreceptor function, was substantially impaired in the *Adipor1* KO mice at 3 weeks of age and gradually progressed thereafter. The b-wave amplitudes of photopic ERGs, which show cone photoreceptor function, were comparable between homozygote *Adipor1* KO and WT and heterozygotes at 3 weeks, while they were decreased in the homozygotes at 28 weeks (Supplementary Fig. 1), indicating that cone dysfunction became evident after rod photoreceptor dysfunction progressed.

### Photoreceptor cell death in *Adipor1* KO mice

**Fig. 3:**
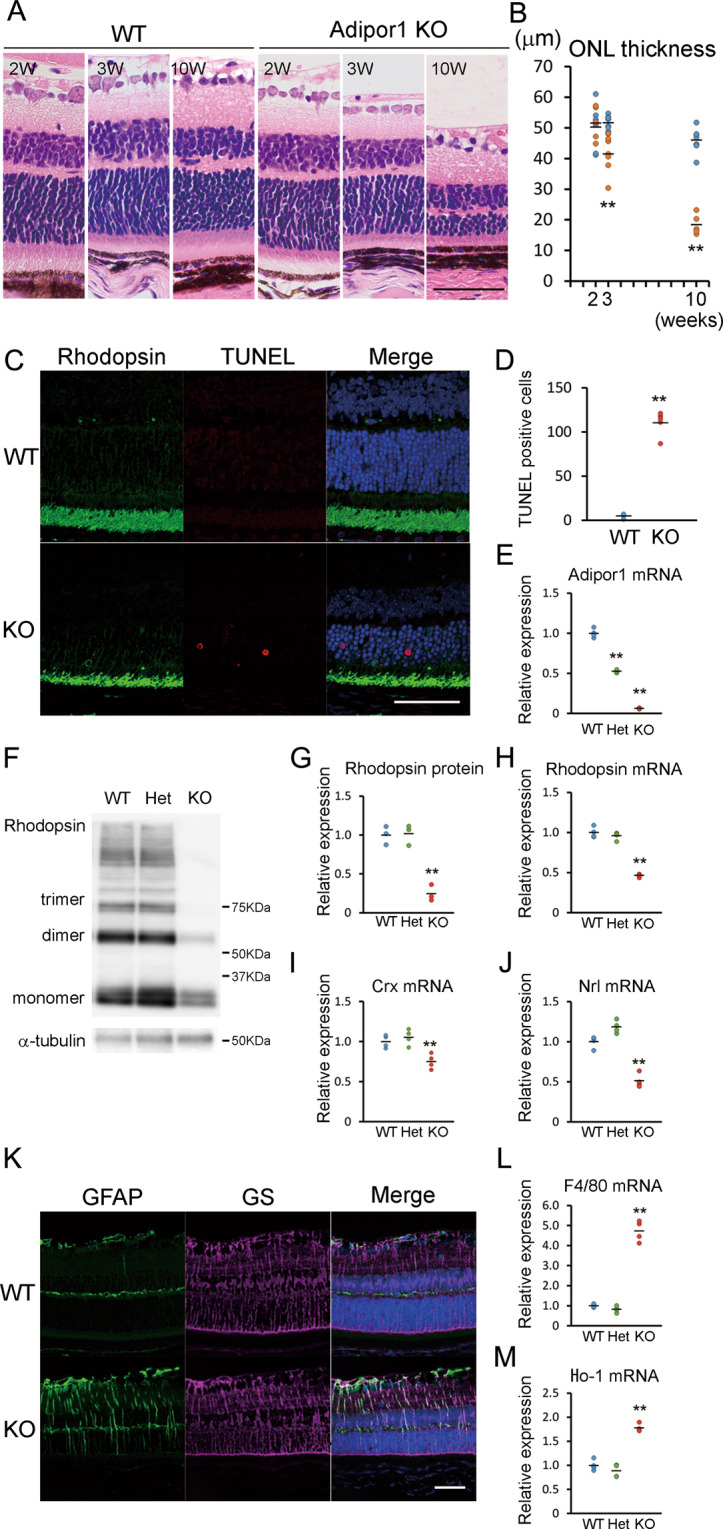
Photoreceptor cell death in homozygous *Adipor1* KO mice. Hematoxylin–eosin (H&E) staining (**A**) of retinal sections, and ONL thickness (**B**) from 2-, 3-, and 10-week-old WT and homozygous *Adipor1* KO mice (**A**). ONL thickness was not different between WT and KO mice at the age of 2 weeks; however, it significantly decreased at 3 weeks, and the difference was further evident at 10 weeks. *n* = 5–9. **C**, **D** TUNEL assay (magenta) with immunohistochemistry staining for rhodopsin (green) in the retina of 3-week-old mice. The number of TUNEL-positive cells increased (**D**), and rhodopsin expression in POS decreased in the homozygous KO retina. n = 5. **E** Real-time PCR. mRNA levels of *Adipor1* in the retina of WT, heterozygous, and homozygous KO mice were confirmed. *n* = 5. **F**, **G** Immunoblot analysis. Rhodopsin protein levels decreased in the homozygous KO mice retina at 3 weeks of age. *n* = 3. **H**–**J** Real-time PCR. mRNA levels of *Rhodopsin* (*Rho)* (H), *Crx* (I), and *Nrl* (J) in the retinas decreased in the homozygous KO mice at 3 weeks of age. (F-J) There were no differences between WT and heterozygotes, *n* = 4. **K** Immunohistochemistry for GFAP (green), a marker for reactive glia, and GS, a marker of Müller glial cells (magenta). GFAP was colocalized with GS and was upregulated in the homozygous KO mice retina at 3 weeks of age. *n* = 5. **L**, **M** Real-time PCR. mRNA levels of F4/80 (**L**) and Ho-1 (**M**) increased in the retina of homozygous KO mice at 3 weeks of age. There were no differences between WT and heterozygotes, *n* = 4. GFAP glial fibrillary acidic protein, GS glutamine synthetase, Het heterozygotes, POS photoreceptor outer segment. Data are shown as means ± standard deviations. **P < 0.01 WT, two-tailed Student’s *t* test in (**B**, **D**), vs. WT, one-way ANOVA in (**E**, **G**, **H**, **I**, **J**, **L**, **M**). Scale bar, 50 μm.

Histological changes in photoreceptors were analyzed using hematoxylin and eosin staining. The thickness of the photoreceptor layer, the ONL, was compared between WT and homozygous *Adipor1* KO mice. At 2 weeks of age, no significant differences were observed; however, ONL thickness was significantly reduced by 3 weeks, and the change was further evident at 10 weeks because of progressive loss of photoreceptors in KO mice (Fig. 3A, B). TUNEL staining revealed more apoptotic cells only in the ONL of the homozygous KO mice at 3 weeks (Fig. 3C, D). Moreover, among WT, heterozygous, and homozygous KO mice (Fig. 3E), the abundance of rhodopsin was already remarkably reduced in the retina of homozygous KO mice at 3 weeks (Fig. 3F, G), while changes were not observed in heterozygotes. Further, *Rhodopsin* (*Rho*) mRNA levels (Fig. 3H), those of its upstream transcription factors, *Crx* (Fig. 3I), *Nrl* (Fig. 3J), and rod photoreceptor markers, *Gnat1* (Supplementary Fig. 2) and *Pde6b* (Supplementary Fig. 2) were repressed in homozygotes. In contrast, the levels of cone photoreceptor markers, *Arr3* (Supplementary Fig. 2) and *Pde6c* (Supplementary Fig. 2) did not change. In homozygous *Adipor1* KO mice, GFAP expression, which represents reactive glia, was increased in Müller glial cells expressing GS, suggesting that Müller glial cells were also affected directly or indirectly by ADIPOR1 deficiency (Fig. 3K). Similarly, F4/80, a macrophage/microglia marker (Fig. 3L), and Ho-1, an oxidative stress marker (Fig. 3M), mRNA expression was increased, suggesting that microenvironmental stress was upregulated in the absence of retinal ADIPOR1.

### DHA reduction in the *Adipor1* KO retina

**Fig. 4:**
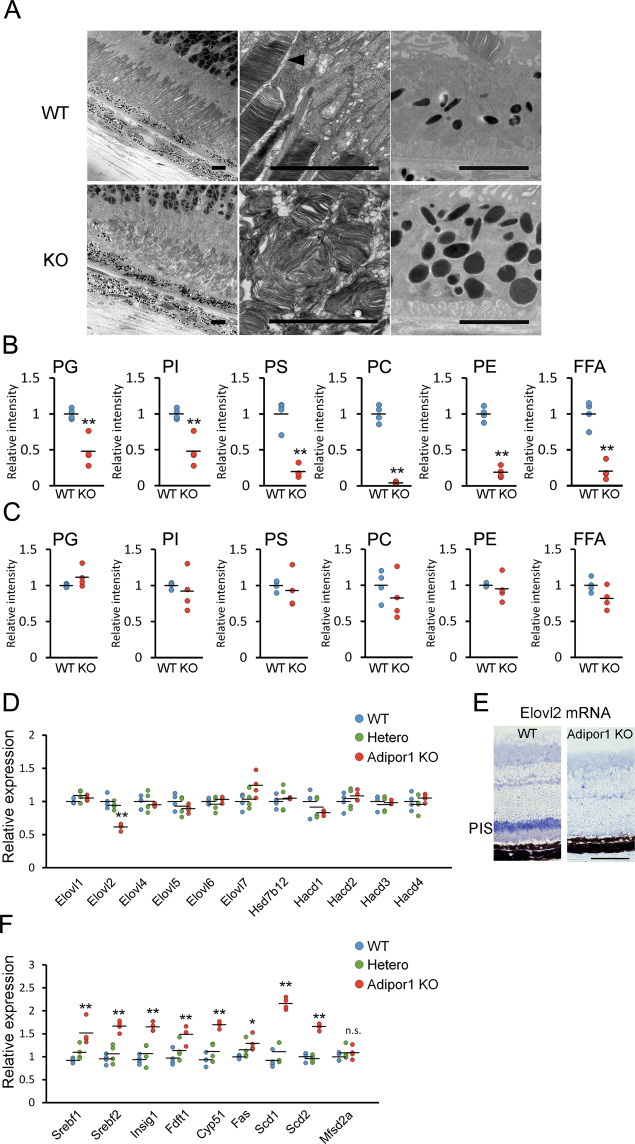
DHA is reduced in *Adipor1* KO retinas. **A** Electron microscopy images of the outer layers of WT and homozygous *Adipor1* KO retinas at 2 weeks of age. Low (left) and high (center and right) magnifications of the images. Photoreceptor outer segment discs, composed of plasma membranes containing phospholipids, were misaligned and melanosomes in the RPE were increased in the *Adipor1* KO retina. (**B**, **C**) LS/MS assay of the WT and *Adipor1* KO retinal samples at 2 weeks of age. Relative levels of DHA containing phospholipids (**B**) and oleic acid-containing phospholipids (**C**). DHA-containing phospholipids were reduced while oleic acid-containing phospholipids were preserved in the retina of KO mice compared with that of WT mice. **D** Real-time PCR. Relative mRNA levels of proteins associated with the fatty acid elongation pathway in the retina at 2 weeks of age. *Elovl2* was repressed in the retina of homozygous KO mice compared with that of WT and heterozygous mice. **E** In situ hybridization. *Elovl2* mRNA was prominently expressed in the PIS and weakly in the inner layers of the WT mouse retina at 2 weeks of age. However, the expression was reduced in the homozygous KO retina. **F** Real-time PCR. Relative mRNA levels of molecules in the lipogenesis pathway in the retina of homozygous KO mice compared to that of WT and heterozygous mice at 2 weeks of age. **D**, **F** There were no differences between WT and heterozygotes. PIS photoreceptor inner segments, RPE retinal pigment epithelium. Data are shown as means ± standard deviations. *n* = 4, *P < 0.05, **P < 0.01 vs. control, two-tailed Student’s *t* test in (**B**), vs. WT, one-way ANOVA in (**D**, **F**). Scale bar, 5 μm (**A**), 50 μm (**E**).

Further analyses were performed in 2-week-old mice to explore the pathogenesis during the development of the above-described phenotypes. The POSs, where folded plasma membranes are regularly aligned and retain rhodopsin as discs, were already misaligned and damaged in the retina of *Adipor1* KO mice (Fig. 4A). Pigmented melanosomes were increased in the RPE, where POSs are phagocytosed and digested to regenerate the visual pigment^12^.

Next, to determine whether disorganized POSs are accompanied by changes in lipid bilayer composition, retinal phospholipids were quantified using (LC–MS). In the retinas of homozygous KO mice, phospholipids containing DHA as FFAs, such as phosphatidylglycerol, phosphatidylinositol, phosphatidylserine, phosphatidylcholine, and phosphatidylethanolamine, were substantially reduced (Fig. 4B), while those containing oleic acid were not affected (Fig. 4C).

Because DHA can be synthesized by a series of enzymatic reactions, the mRNA expression of enzymes that participate in fatty acid elongation was quantified. *Elovl2* mRNA was downregulated (Fig. 4D), while those of other enzymes, such as of *Elovl4*, were not. This change was observed only in homozygotes, not heterozygotes. Moreover, *Elovl2* mRNA was predominantly expressed in the PISs and in the inner layers of the retina (Fig. 4E). Consistent with the real-time PCR results (Fig. 4D), *Elovl2* was suppressed in the PIS of homozygous *Adipor1* KO mice (Fig. 4E).

The expression of genes that contribute to lipogenesis in the retina was further analyzed. In the homozygous KO mouse retina, mRNA expressions of the transcription factors *Srebf1* and *Srebf2*, which regulate lipid synthesis, as well as their downstream genes, were increased (Fig. 4F). However, no change was observed in *Mfsd2a* expression, the deficiency of which causes retinal degeneration via decreased DHA trafficking^27^.

### Adipor1 KD represses Elovl2 in vitro

**Fig. 5:**
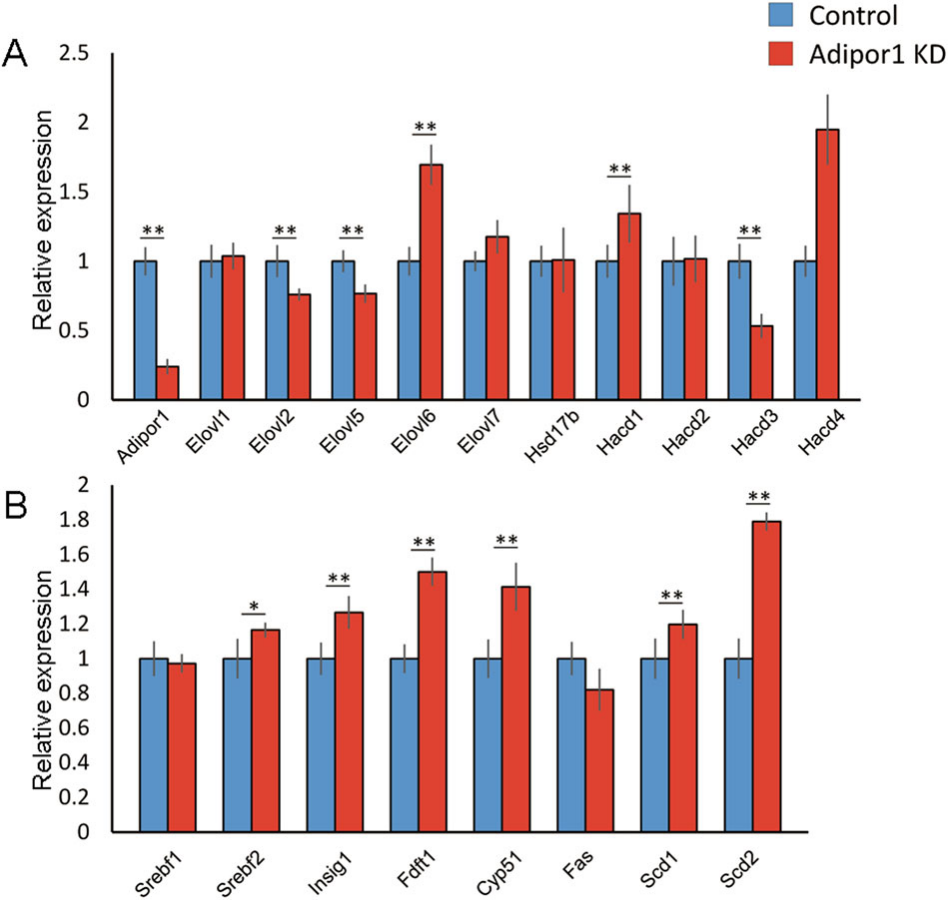
*Elovl2* repression by *Adipor1* knockdown in vitro. **A**, **B** Real-time PCR. *Aidpor1* knockdown using siRNA in bEnd.3 cells was confirmed by real-time PCR (**A**). Under the same condition, *Elovl2* wasrepressed (**A**), and lipogenesis-associated pathway mRNAs were upregulated (**B**). Data are shown as means ± standard deviations. *n* = 10, *P < 0.05, **P < 0.01 versus negative control, two-tailed Student’s t-test.

*Adipor1* KD decreased *Elovl2* mRNA levels in bEnd.3 cells, indicating that *Elovl2* transcription was, at least in part, regulated by ADIPOR1 (Fig. 5A). Similarly, *Elovl5*, which also elongates polyunsaturated acyl-CoA, and *Elovl2*^28^ were also downregulated, whereas *Elovl6*, which acts on saturated acyl-CoA, and 3-hydroxy acyl-CoA dehydrogenase 1 and 4^29^ were upregulated. In addition, *Srebf2* and its downstream genes related to lipogenesis were increased (Fig. 5B) similar to the in vivo observations.

CpG island of the upstream of *Elovl2* gene promoter region was analyzed using bisulfite PCR to evaluate the changes of DNA methylation status with or without *Adipor1* KD. There were several points where methylation status was significantly different after *Adipor1* KD (Supplementary Fig. 3).

## Discussion

*Adipor1* mRNA was found to be expressed throughout the retina and prominently in the PIS. Its level increased in the retina after birth until 3 weeks of age. Visual function was impaired in homozygous *Adipor1* KO mice as early as at 3 weeks, at which point rhodopsin expression was substantially reduced, while apoptosis-induced photoreceptor loss was starting. Electron microscopy images revealed that misalignment of the POS, the location where rhodopsin is retained, was already present at 2 weeks of age. Moreover, DHA-containing phospholipids were reduced and *Elovl2* was suppressed by 2 weeks. The reduced expression of *Elovl2* by ADIPOR1 deficiency was also confirmed using an in vitro KD system.

*Adipor1* mRNA expression gradually increased from immediately after birth to postnatal 3 weeks, which corresponded to photoreceptor development and maturation. The photoreceptor connecting cilium, through which all POS proteins and membrane components must be conveyed, appears between the PIS and POS at P3 and is developed by 2 weeks^30^. A previous study reported the presence of ADIPOR1 protein in the POS^31^, and the current study showed that the increase in *Adipor1* expression was parallel to POS development. Considering that *Adipor1* mRNA was concentrated in PISs, where organelles such as ribosomes and the Golgi apparatus are accumulated^32^, the ADIPOR1 protein may be formed in PISs and transferred through the connecting cilium to POSs during their development.

The a-wave amplitude in ERG was substantially decreased in *Adipor1* KO mice compared to WT mice at 3 weeks of age, consistent with the finding of previous reports analyzing *Adipor1* KO mice from another lineage (*AdipoR1*^gt^)^16^, indicating impaired photoreceptor function in the absence of ADIPOR1. However, while ONL thinning was mild at this time point, and it progressed rapidly thereafter, the photoreceptor function was substantially reduced by 3 weeks and did not remarkably progress afterward. Considering that rhodopsin mRNA and protein levels, together with its upstream transcription factors, *Crx* and *Nrl*, were substantially decreased by 3 weeks, reduced rhodopsin expression in the POSs of live photoreceptors may have more significantly contributed to impaired photoreceptor responses, than apoptosis-induced photoreceptor loss at this time point. Rhodopsin suppression was consistent with the findings of a previous study reporting that rhodopsin protein abundance was reduced in the retina of systemic *Adipor1* KO mice from another lineage (*Adipor1* < tm1.2Lex > )^31^ and of *Adipor1* KD by adenovirus^31^; there was a possibility that rhodopsin reduction was due to protein degradation related to POS degeneration by reduction in DHA, one of the major components of POS discs^12,13^. However, the current study extended the results to describe transcriptional repression, which was not previously defined; thus, rhodopsin was reduced as a result of impaired production. The influence on rhodopsin in *Adipor1* KO mice was also in contrast to ciliopathy, a congenital disease in which no POSs develop, and associated with ectopic rhodopsin expression due to impaired rhodopsin trafficking^33^; *Adipor1* KO mice, which also showed POS deficiency, did not exhibit ectopic rhodopsin expression and simply showed rhodopsin repression.

Similar to that observed in *Adipor1* KO mice in the current study, photoreceptor apoptosis has also been previously reported to start at 3 weeks of age in *Rhodopsin (Rho)* KO mice^34^. Given that the neuronal survival pathway, insulin-phosphoinositide 3-kinase signaling, can become activated by rhodopsin-mediated visual signals^35^, rhodopsin suppression caused by ADIPOR1 deficiency may have accelerated photoreceptor apoptosis, although this requires further investigation.

In addition, Müller glial cells, which preserve homeostasis of the tissue microenvironment^36,37^, became reactivated to increase GFAP expression, and macrophage and oxidative stress markers in the retina were increased in the *Adipor1* KO mice. These results suggest that the apoptotic and degenerative changes in the photoreceptors may have caused inflammation and oxidative stress^38^, which subsequently affected the entire retina, although a direct effect of ADIPOR1 deficiency on these cells could not be excluded. However, the whole retinal reaction related to photoreceptor degeneration is consistent with the results of previous reports with retinal inflammation^39,40^ and light exposure-induced oxidative stress^41^ models. Thus, inflammation and oxidative stress may have accelerated neurodegeneration in the retina of *Adipor1* KO mice.

Cone system dysfunction was not observed, and the expression of cone markers did not change at the age of 3 weeks, while the dysfunction became evident by 28 weeks in the *Adipor1* KO mice, suggesting that the cone system abnormality had gradually progressed and became evident later than rod photoreceptor degeneration. It has been reported that rod-derived cone viable factor (RdCVF), which is a soluble factor released from rod photoreceptors that regulates glucose metabolism^42^ and oxidative stress^43^ in the cone photoreceptors, is indispensable for cone photoreceptor function^44^. Delayed cone photoreceptor dysfunction may have been, at least partly, related to rod photoreceptor degeneration in the current study.

A previous report described reduced uptake of labeled DHA in the retina of *Adipor1* KO mice at 3 weeks of age, at which point the photoreceptors were already reduced only in the homozygotes^16^. However, the labeled DHA taken up by the eye-cup tissue was also substantially reduced in the heterozygous tissue, while total DHA was restored in heterozygote retinas, and the amount of DHA taken up was low compared to the total DHA levels in the retina (approximately 1:10,000 according to the previous report)^16^. The amount of DHA delivered via the circulation and taken up by the RPE to subsequently supply photoreceptors was reduced by ADIPOR1 deficiency. However, an alternative system associated with ADIPOR1 signaling is likely to present, potentially in photoreceptors, that serves to regulate total DHA levels within the retinal tissue, which may act independently of the circulating DHA system.

In the current study, we found that the fatty acid elongation enzyme, ELOVL2, which elongates C20–C24 PUFAs, such as arachidonic acid (20:4n-6), eicosapentaenoic acid (20:5n-3), docosatetraenoic acid (22:4n-6), and docosapentaenoic acid (22:5n-3)^18,45^, was suppressed in the retina of homozygous, but not heterozygous, *Adipor1* KO mice, at the age of 2 weeks before the time point when the photoreceptor loss was evident. Moreover, ELOVL2 was downregulated by *Adipor1* KD in vitro. In addition, *Elovl2* mRNA was predominately expressed in PISs, similar to that observed for *Adipor1*. Taken together, these results suggest that ADIPOR1 may affect *Elovl2* transcription, thereby interfering with DHA production via elongation of the carbon chain by substrates, such as γ-linolenic acid, in the retina. *Elovl2* expression was not reduced in the retinas of heterozygotes, consistent with the total DHA levels, which were not affected in the heterozygous retina. Moreover, the absence of *Elovl2* repression in heterozygotes was consistent with the absence of photoreceptor dysfunction in *Adipor1* heterozygotes.

In fact, *Elovl2* KO caused reduced DHA levels in the liver and serum, indicating that DHA can be provided endogenously through ELOVL2 action, not only derived from the diet^45^. High-fat diets do not induce hepatic steatosis^45^, and impaired spermatogenesis related to DHA insufficiency is not rescued by DHA supplementation, both in *Elovl2* KO mice^46^, suggesting that endogenously synthesized DHA is essential for lipid homeostasis in specific tissues and organs^28,45^. Moreover, *Elovl2* mutant mice, in which Elovl2 enzymatic activity was reduced, showed decreased DHA contents in the retina and visual dysfunction of rod photoreceptor cells^47^ at 6 months of age. Thus, *Elovl2* deficient mice show similar retinal phenotypes to *Adipor1* KO mice when they age. Deficiency in endogenously synthesized DHA activates hepatic SREBP-1c, which is translated from *Srebf1* mRNA, and stimulates transcription of lipogenic genes^45^. Therefore, the upregulation of lipogenic genes in the retina of *Adipor1* KO mice, as well as in vitro, was consistent with the condition described above for ELOVL2 deficiency^45^. One known regulatory system in *Elovl2* transcription is the changes in DNA methylation^47–50^. In fact, several parts of the CpG island showed different levels of DNA methylation after *Adipor1* KD in vitro in the current study. Future study to analyze whether the changes promote the repression of Elovl2 is warranted. Alternatively, ADIPOR1 is required for the expression of desaturase, which acts upstream of ELOVL2 during DHA synthesis^51^; ADIPOR1 deficiency may have decreased desaturase, thereby reducing the substrate of ELOVL2 and *Elovl2* expression. The mechanisms underlying the connection between *Adipor1* and *Elovl2* would be an area of interest for future research.

Finally, the expression of a DHA transporter, *Mfsd2a*, was not altered in the retina of *Adipor1* KO mice, although pigmented melanosomes were increased in the RPE of *Adipor1* KO mice similar to that in *Mfsd2a* KO mice^52^. However, *Mfsd2a* KO mice do not exhibit photoreceptor degeneration^52^, supporting the notion that photoreceptor degeneration is not induced by a reduction in DHA delivery, but rather by reduced endogenous DHA production via the fatty acid elongation system.

In conclusion, ADIPOR1 deficiency-induced reduced expression of *Elovl2*, a fatty acid elongation enzyme that produces DHA in local tissues. Decreased DHA in the retinas of *Adipor1* KO mice likely involved reduced DHA production in the photoreceptors through the ELOVL2 enzymatic reaction, which subsequently caused photoreceptor damage and visual impairment. The finding will help explore a new therapeutic approach for treating retinal degeneration induced by DHA depletion due to ADIPOR1 deficiency in the future.

## Availability of data and materials

The datasets generated or analyzed during the current study are available from the corresponding author on reasonable request. The data have also been uploaded as Supplementary Information.

## Supplementary information

Supplementary Figure Legends

Supplemental Figure 1

Supplemental Figure 2

Supplemental Figure 3

data set
