# Supplementary material for: ADIPOR1 deficiency-induced suppression of retinal ELOVL2 and docosahexaenoic acid levels during photoreceptor degeneration and visual loss
Source: Cell Death Dis. 2021 May 7;12(5):458. doi: 10.1038/s41419-021-03741-5 (PMC8105316; doi:10.1038/s41419-021-03741-5)
Supplement: Supplementary file 1 — Supplementary Figure Legends [file 41419_2021_3741_MOESM1_ESM.doc]

**Supplementary Figure 1. Photopic ERGs of *Adipor1* KO mice.**

Photopic ERGs at 3 (A), and 28 (B) weeks of age. Representative wave form from individual mice at each stimulus intensity (A and B). The b-wave amplitudes of homozygous KO mice were comparable to those of WT and heterozygous mice at 3 weeks; however, their reduction was evident at 28 weeks. No differences were observed between WT and heterozygote mice. Data are shown as means ± standard deviations. n = 5 (all male) for data at 3 weeks, n of WT, 4 (male 4, female 2); heterozygotes, 6 (male 4, female 2); homozygotes, 6 (male 3, female 3) for data at 28 weeks. **P < 0.01 versus WT, one-way ANOVA.

**Supplementary Figure 2. Photoreceptor markers in the retina of *Adipor1* KO mice.**

Real-time PCR. Rod photoreceptor markers, *Gnat1* and *Pde6b*, were decreased; however, cone photoreceptor markers, *Arr3* and *Pde6c*, did not change in the retina of homozygous *Adipor1* KO mice compared with heterozygote and WT mice at 3 weeks of age. n = 4, **P < 0.01 versus c WT, one-way ANOVA.

**Supplementary Figure 3. Methylation analysis with or without *Adipor1* knockdown *in vitro*.**

(A, upper) Schematic representation of the sequence of the CpG island of *Elovl2* gene. Blue box indicates exons of *Elovl2* Gene. DNA methylation status of 96 CpG sites of *Elovl2* gene promoter region was analyzed using bisulfite PCR after control or *Adipor1* siRNA introduction to bEnd.3 cells. (A, lower) Each row corresponds to one representative clone. Methylated CpG sites are marked as filled circles and unmethylated sites as open circles. (B) Methylation status of the whole CpG island in bEnd.3 cells analyzed by QUMA program. The ratio (%) of methylated clones in each CpG site. There were several points where methylation status was significantly different after *Adipor1* KD. n for Control, 30 clones; *Adipor1* KD, 35 clones. * P < 0.05, **P < 0.01, Fisher’s exact test.
