# Supplementary figures and images for: ADIPOR1 deficiency-induced suppression of retinal ELOVL2 and docosahexaenoic acid levels during photoreceptor degeneration and visual loss
Source: Cell Death Dis. 2021 May 7;12(5):458. doi: 10.1038/s41419-021-03741-5 (PMC8105316; doi:10.1038/s41419-021-03741-5)

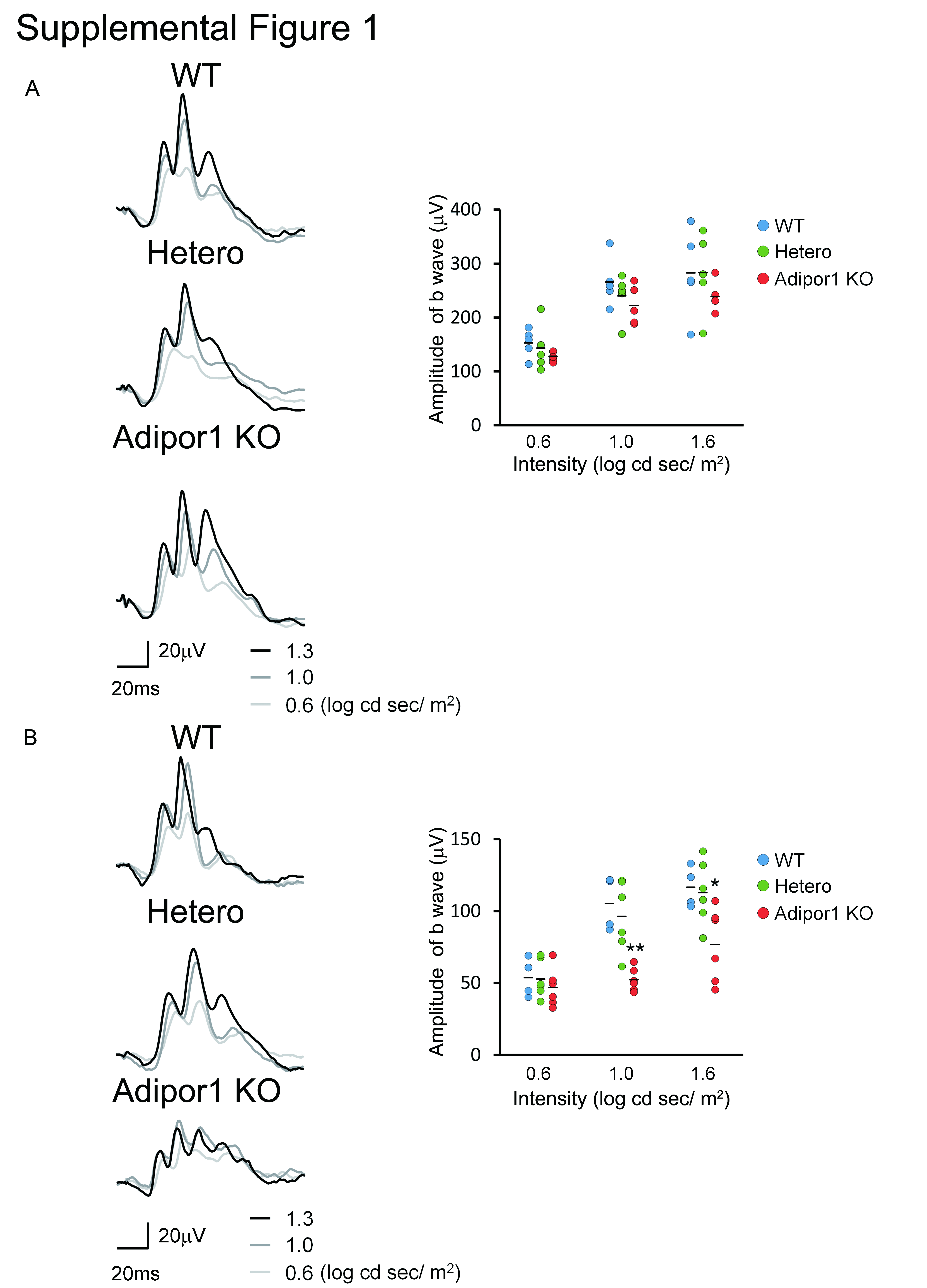

Supplement: Supplementary file 2 — Supplemental Figure 1 [file 41419_2021_3741_MOESM2_ESM.tif]

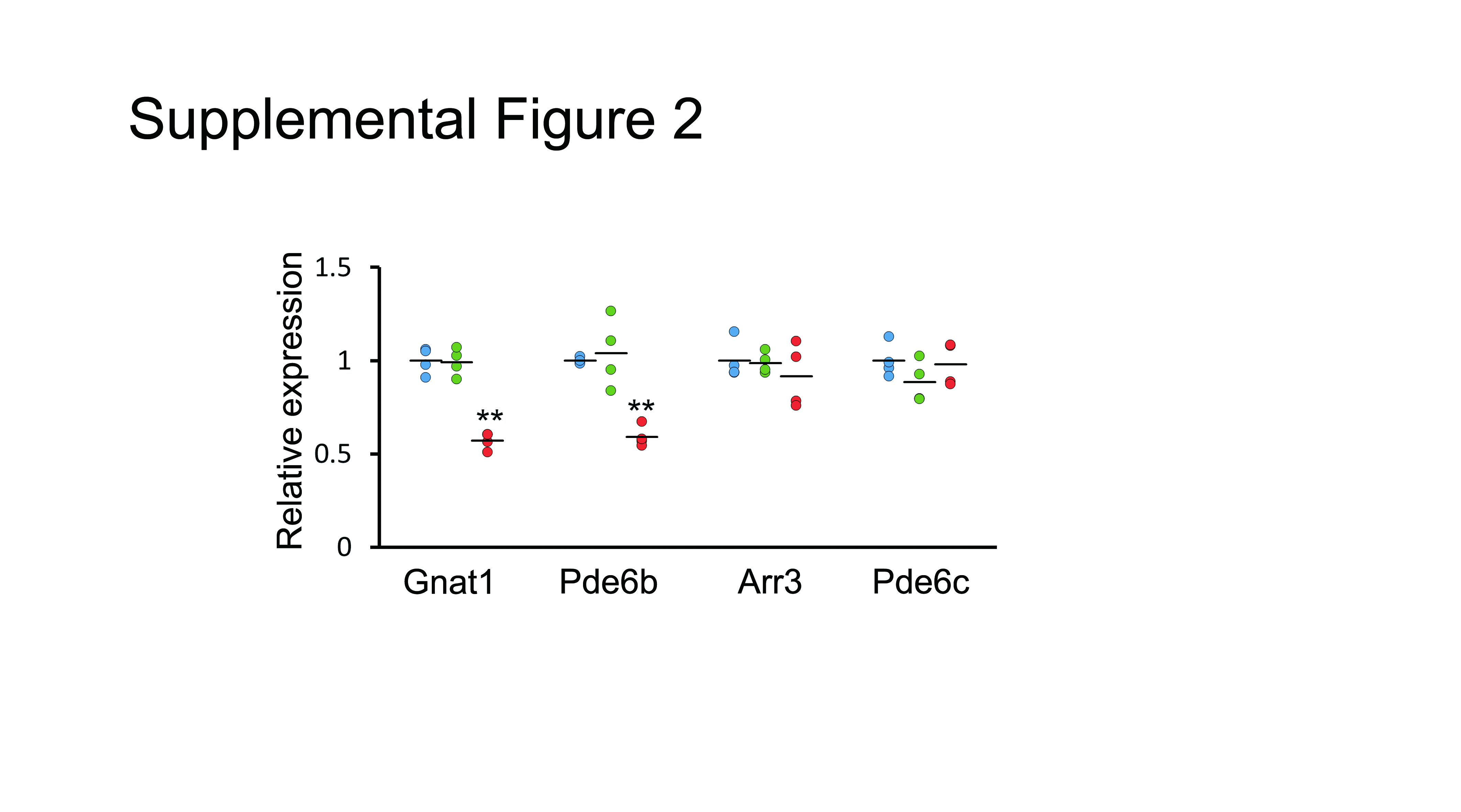

Supplement: Supplementary file 3 — Supplemental Figure 2 [file 41419_2021_3741_MOESM3_ESM.tif]

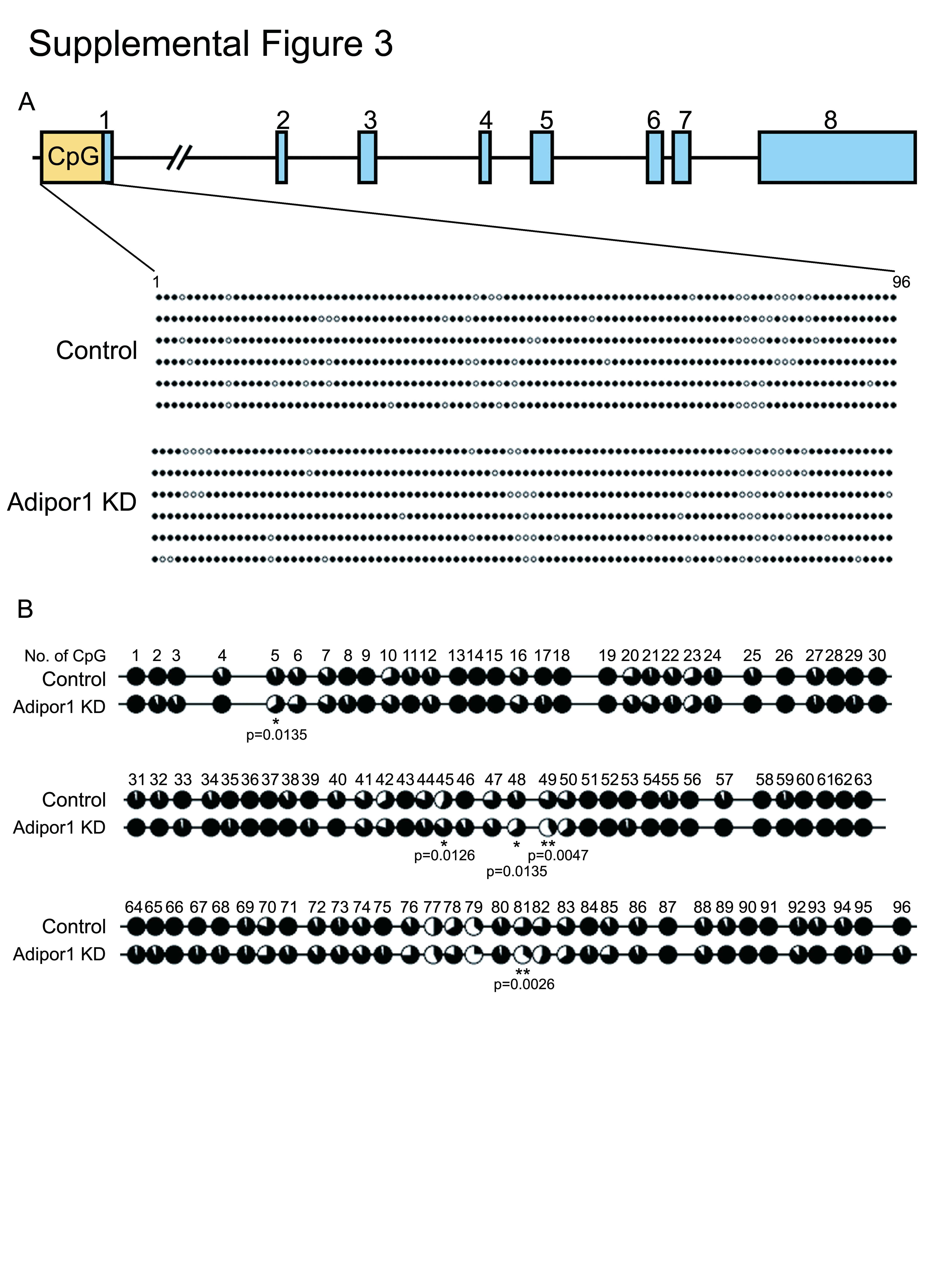

Supplement: Supplementary file 4 — Supplemental Figure 3 [file 41419_2021_3741_MOESM4_ESM.tif]
